# Supplementary material for: Emission enhancement of erbium in a reverse nanofocusing waveguide
Source: Nat Commun. 2023 May 11;14:2719. doi: 10.1038/s41467-023-38262-6 (PMC10175264; doi:10.1038/s41467-023-38262-6)
Supplement: Supplementary file 1 — Supplementary Information [file 41467_2023_38262_MOESM1_ESM.pdf]

# Supplementary Information: Emission enhancement of erbium in a reverse nanofocusing waveguide

*Nicholas A. Güsken*[*,1,2], Ming Fu[1], Maximilian Zapf[3], Michael P. Nielsen[1,4], Paul Dichtl[1], Robert Röder[3], Alex S. Clark[1,5], Stefan A. Maier[1,6], Carsten Ronning[3] and Rupert F. Oulton[*,1]*

*[1]Department of Physics, Imperial College London, Prince Consort Road, London SW7 2AZ, UK*

*[2]Department of Materials Science and Engineering, Stanford University, Stanford, CA 94305, USA*

*[3]Friedrich-Schiller-Universität Jena, Max-Wien-Platz 1, 07743 Jena, Germany*

*[4]School of Photovoltaics and Renewable Energy Engineering, UNSW Sydney, Kensington, NSW 2052, Australia*

*[5]Quantum Engineering Technology Labs, University of Bristol, BS8 1UB, UK*

*[6]Monash University School of Physics and Astronomy, Clayton, VIC 3800, Australia*

## 0. Contents

0. Contents ... 1
1. Excitation field enhancement & polarization dependence ... 2
2. Device fabrication ... 3
3. Hybrid plasmonic gap waveguide: Propagation length and coupling efficiency ... 4
4. Luminescence background ... 9
5. Rate equation model and the Purcell factor $F_P$ in saturation regime ... 10
6. Gaussian saturation model and Purcell enhancement results ... 16
7. Polarization dependence of pump beam to waveguide coupling ... 20
8. Signal collection efficiency ... 22
9. Purcell enhancement waveguide coupling of electric-dipole (ED) and magnetic-dipole (MD) emission ... 24
10. Experimental setup ... 24
11. Luminescence lifetime measurements ... 25
12. Power dependence of the signal spectrum ... 29

## 1. Excitation field enhancement & polarization dependence

The pump beam, which excites the erbium ions in the metal-insulator-metal (MIM) gap from the top, is linearly polarized. The coupling of pump power into the gap strongly depends on the pump beam's polarization axis with respect to the waveguide's orientation, i.e. perpendicular to the gap vs. parallel to the gap. This is due to the fact that the fundamental mode of the MIM structure under study is transverse electric (TE), with field lines perpendicular to the longitudinal orientation of the gap (c.f. Figure S1 a1). Hence, solely TE (perpendicular) polarized light couples to the fundamental gap mode as illustrated in Figure S1. This has been studied in the literature[1,2] and is specifically shown here for a $w = 10$ nm gap waveguide in the structure studied in the main manuscript.

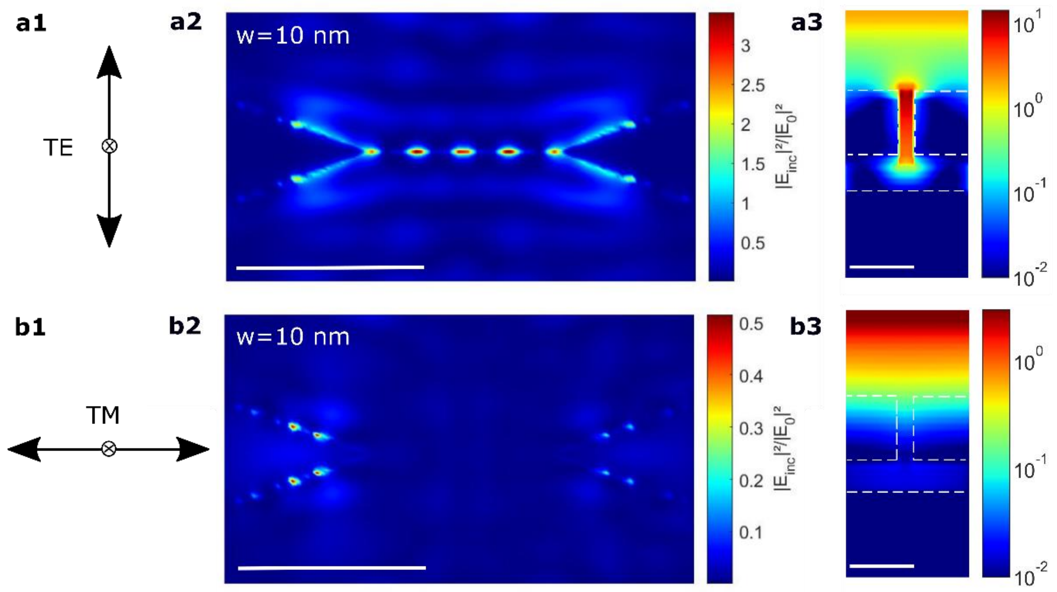


**Figure S1| Polarization dependence of a 10 nm wide gap waveguide at 980 nm pump wavelength**. **a1**, TE polarization of the pump beam with a perpendicular field direction with respect to the longitudinal gap. **a2**, 3D FDTD simulation showing the normalized field intensity cross-section $|E_{inc}|^2/|E_0|^2$ at a depth of 1 nm underneath the gap waveguide for a waveguide system with $w = 10$ nm gap width (horizontal monitor). Scale bar 1 µm. **a3**, 3D FDTD simulation showing the normalized field intensity cross-section $|E_{inc}|^2/|E_0|^2$ (log-scale) as a side view of the gap waveguide system for a 10 nm gap (vertical monitor). Scale bar 50 nm, the white dotted lines indicate the contours of the Au gap and the Si-slab substrate, respectively. The spacer interlayer is $SiO_2$. **b1 - b3**, same simulations as in **(a2)** and **(a3)** but with a TM polarized pump beam. All intensity values are normalized with respect to the same incident pump intensity.

Figure S1 a2 and b2 show 3D FDTD simulations of the electrical field intensity at the position of the ions, 1 nm underneath the waveguide for TE and TM, respectively. Weak backscattering at both ends of the waveguide results in a lateral eigenmode resonance (standing wave pattern) of the waveguide with a field enhancement of about 2 on average. This can be only observed for the TE case, in stark

contrast to the TM case for which the field intensity in the gap is negligible. This is underpinned by the vertical cross-sections shown in Figure S1 a3 and Figure S1 b3, illustrating the electric field intensity in the gap waveguide for TE and TM pump polarization, respectively. The ratio of pump field intensity in the gap for the two polarizations varies by about two orders of magnitude. Thus, rotating the pump polarization relative to the gap, essentially allows the luminescence from ions in the gap to be switched on and off.

Please note that the interference pattern observed in Figure S1 a1 stems from weak back reflections at both ends of the waveguide induced by 980 nm excitation light scattered into the waveguide mode. The reflectivity can be estimated to be about 6% based of the intensity distribution of the simulated gap field in Figure S1 a1. Here, $I = |a_{gap}e^{ikz} + a_{gap}re^{-ikz}|^2$, with the amplitude, $a_{gap}$, the reflection coefficient $r$ and propagation constant $k$. Meanwhile the intensity maxima and minima from Figure S1 a1, $I_{max} \approx 3.3$ and $I_{min} \approx 1.2$ yield the average signal intensity of 2.25. Re-writing the intensity expression to $I_{\pm} = \; a_{gap}{}^2\,(1 + r^2) \pm 2a_{gap}{}^2 r$, then the average intensity $a_{gap}{}^2\,(1 + r^2) = 2.25$ and its variation $2a_{gap}{}^2 r = 1.05$. Re-writing and eliminating $a$ yields $\frac{1.05}{2r}(1 + r^2) = 2.25$ from which we can estimate the reflectivity $r^2 \approx 6\%$.

## 2. Device fabrication

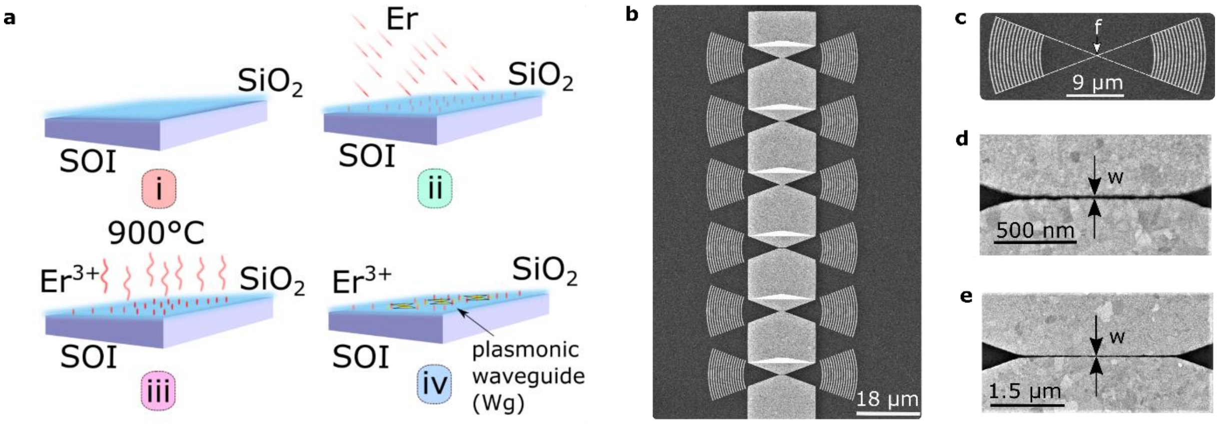


**Figure S2| Fabrication and top views of the erbium doped hybrid gap plasmonic waveguides. a**, i)-iv) Illustration of the individual fabrication steps of the erbium doped Wg reverse nanofocusing platform. **b,** Array of gold waveguides with different gap widths and focusing gratings on both sides. **c,** Image of the “grating-to-grating” (Gr-Gr) device explained in the main text. The white dotted line overlay highlights the focal position f. **d,** 1 µm long gap waveguide with a gap width of $w = 10 \pm 3$ nm. **e,** 3 µm long gap waveguide with a gap width of $w = 12 \pm 3$ nm.

An outline of the individual fabrication steps of the reverse nanofocusing hybrid gap plasmonic waveguides (Wg) with implanted $Er^{3+}$-ions is illustrated in Figure S2a while top view SEM images of the resulting structures are depicted in Figure S2b – e. First, a $25\ \mathrm{nm}\ \mathrm{SiO_2}$ layer was deposited onto industry standard silicon-on-insulator (SOI) substrates. Then, erbium ions were implanted into the $\mathrm{SiO_2}$ interlayer using ion implantation with an acceleration voltage of $10\ \mathrm{keV}$, a sample tilting angle

of 45° and a fluence of $\rho = 1 \times 10^{15}\ \mathrm{cm}^{-2}$. This resulted in a mean ion implantation depth of $(6.0 \pm 2.5)\ \mathrm{nm}$, as simulated with the Monte-Carlo software package TRIM[3,4] and considering sputtering effects, as shown in Figure S2. After that, the samples were annealed under controlled atmosphere for one hour at $900\ °\mathrm{C}$. Subsequent annealing primarily targets defect removal and optical activation of the implanted centres; however, can also cause diffusion of the implanted atoms towards the implantation surface as reported in the literature[5]. Here, we estimate a maximum mean diffusion length of about $\langle y \rangle = 2\sqrt{D\,t} = 3.8\ \mathrm{nm}$, based on a diffusion coefficient of $D = 10^{-17}\mathrm{cm}^2\mathrm{s}^{-1}$ at 900°C for Er in $SiO_2$. Typically this broadens the depth distribution; however, a preferential diffusion towards the surface can also occur, due to the large density of implantation defect states in the penetrated layer compared to the underlying $SiO_2$[5,6]. This effect results in a re-distribution of the implanted ions and a profile, which is closer to the surface than the simulated TRIM profile (Fig. S3). After annealing, which optically activates erbium and results in $\mathrm{Er}^{3+}$-ions, hybrid gap plasmon waveguides (Wg) were patterned on top of the $\mathrm{Er}^{3+}$-doped $\mathrm{SiO}_2$ interlayer. To produce narrow metal-insulator-metal (MIM) gaps, a two-step EBL process was used, resulting in gaps as small as $10\ \mathrm{nm}$. After each EBL step, a $50\ \mathrm{nm}$ Au layer was deposited via thermal evaporation at a pressure $< 5 \times 10^{-7}\ \mathrm{Torr}$. Finally, the samples were covered with a thick poly(methyl methacrylate) (PMMA) cladding layer.

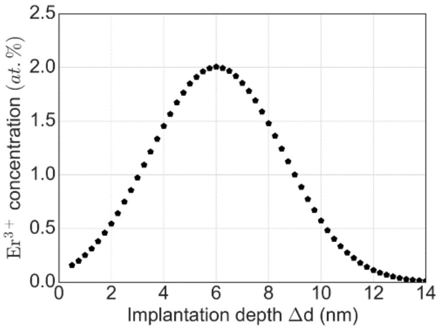


**Figure S3| Implantation depth profile of $Er^{3+}$- ions in a $SiO_2$ interlayer before diffusion (before annealing).** Fluence $\rho = 1 \times 10^{15}\ cm^{-2}$.

## 3. Hybrid plasmonic gap waveguide: Propagation length and coupling efficiency

To accurately estimate i) the propagation length of the hybrid plasmonic mode as well as ii) the coupling efficiency from light exciting the plasmonic gap to the silicon slab waveguide mode, a passive waveguide transmission measurement was performed[7,8]. Here, a CW focused laser beam was coupled in via one grating as e.g. shown in Figure S2b and coupled out via the opposite grating after propagating through the plasmonic gap structure. The input and output light was incident and

collected via the same objective, respectively. However, an iris positioned in the image plane after the objective (c.f. Figure S13) allowed to isolate the signal from the out-coupling grating from the rest of the sample. The input beam was incident under a slight angle created by coming in off-axis through the objective. The in ($R_{cts,in}$) and output ($S_{cts,out}$) signals have been measured (corrected for the background signal as explained below) for various devices of different gap widths in dependence on waveguide length. Based on this information, the system's coupling and guiding efficiency comprising (grating in-coupling, Si-waveguide to plasmonic waveguide, plasmonic propagation, plasmonic waveguide to Si-waveguide and grating outcoupling efficiency) was estimated in dependence on the plasmonic waveguide gap length $l$. This is reflected in $\eta_{meas}(l)$ i.e., Eq. S3.1 and Eq. S3.2 and shown in Figure S4 and Figure S5.

We experimentally and theoretically show that our optimized reverse nanofocusing platform provides coupling efficiencies between gap mode and silicon slab mode of about 80% and propagation lengths of several $\mu$m in the most confined state. The system is identical to the Wg system as described in the main text but without implanted ions. Additionally, grating pitch and duty cycle for the measurement in the main manuscript have been optimized for a central wavelength of 1536 nm.

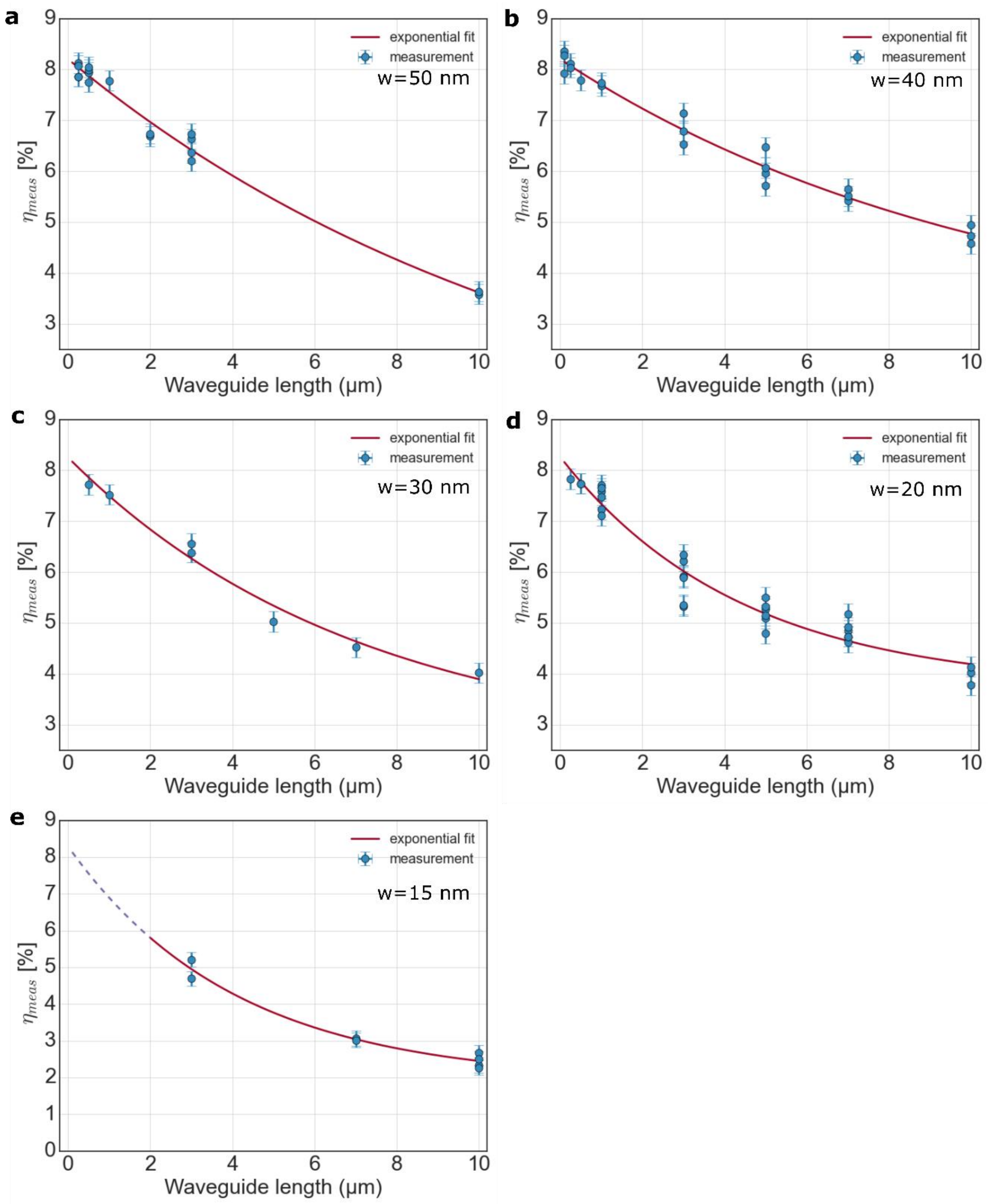


**Figure S4| Waveguide (Wg) cut-back measurements and coupling efficiencies** (Gr-waveguide-Gr) for waveguide widths $w$ of **(a)** $w = 50$ nm, **(b)** $w = 40$ nm, **(c)** $w = 30$ nm, **(d)** $w = 20$ nm and **(e)** $w = 15$ nm. The Power was kept constan for all measurements. From fitting $\eta_{meas} = A_0 + \eta_0 \cdot e^{-L/L_m}$, the following values were extracted for $\eta_{meas}$(lL= 0) = $A_0 + \eta_0$: **(a)** $A_0$ = 0 % + 1.6 % , $\eta_0$ = 8.2 % ± 1.6 %, $L_m$ = 12.3 μm ± 3.6 μm; **(b)** $A_0$ = 2.6 % ± 1.1 %, $\eta_0$ = 5.6 % ± 1.0 %, $L_m$ = 10.5 μm ± 3.0 μm; **(c)** $A_0$ = 2.8 % ± 1.2 %, $\eta_0$ = 5.8 % ± 1.0 %,, $L_m$ = 7.1 μm ± 2.7 μm; **(d)** $A_0$ = 3.7 % ± 0.34 %, $\eta_0$ = 4.5 % ± 0.3 %, , $L_m$ = 4.4 μm ± 0.8 μm; **(e)** $A_0$ = 1.91 % ± 0.6 %, $\eta_0$ = 6.3 % ± 1.1 %, $L_m$ = 4.1 μm ± 1.7 μm. Data points in all panels are the mean and error bars the standard deviation.

The propagation length of the Wg mode for varying gap width, w, can be evaluated via the so-called *cut-back* method[8]. The in- and out-coupled signal of the entire system is compared for waveguides of constant widths $w$ but different lengths $L$, as shown in Figure S4. The detected signal, $\eta_{meas}$, for the different waveguide lengths can be approximated by an exponential decay law of the plasmonic mode in the waveguide, $\eta_{meas} \propto \eta_0 e^{-L/L_m}$ with the initial amplitude being $\eta_0$ and the fundamental

mode's propagation length $L_m$. This method possesses the great advantage that $L_m$ can be determined independently of the respective taper and grating efficiencies. The extracted propagation lengths as a function of gap width are shown and compared to theory in Figure S4a.

We define $\eta_{meas}$ as the ratio between the in- and out-coupled signal via the gratings, measured using a Peltier-cooled IR camera at constant integration time, integration area and pump power of 6.6 µW. The incident light, coupled to the waveguide via the in-coupling grating, and from the waveguide to the out-coupling grating was evaluated via the signal, $S_{cts,out}$, emitted from the grating. This value is normalised by a reference signal, $R_{cts,in}$, collected in reflection from a thick, fully reflecting Au patch over the same area and at the same power:

$$\eta_{meas} = \frac{S_{cts,out} - B_{cts,out}}{R_{cts,in} - B_{cts,in}} . \tag{S3.1}$$

The signal and pump counts have been corrected by the background signals, $B_{cts,out}$ and $B_{cts,in}$, respectively. During the measurement, an iris positioned in the image plane after the objective (c.f. Figure S13) allowed us to isolate the signal from the out-coupling grating spatially from the rest of the sample.

The efficiency measurements shown in Figure S4 provide an estimate for the in-coupling efficiency $\eta_{inc,gap}$ into the plasmonic gap waveguide at ($L = 0$). This has been extracted from the measurements using:

$$\eta_{meas}(l = 0) = \eta_{inc,gap}\eta_{outc,gap}\eta_{Gr-Gr} \Leftrightarrow \eta_{inc,gap} = \sqrt{\frac{\eta_{meas}(L = 0)}{1.1 \cdot \eta_{Gr-Gr}}} , \tag{S3.2}$$

where $\eta_{outc,gap}$ is the out-coupling efficiency. $\eta_{Gr-Gr} \approx 9\%$ is the grating-to-grating coupling efficiency, which we were able to measure directly using a control sample without the metal taper shown in Figure S2c. At first glance, one might assume that the focusing and de-focusing efficiencies would be approximately the same, $\eta_{inc,gap} \approx \eta_{outc,gap}$. We have checked this by simulations (3D FDTD) to find that $\eta_{outc,gap}/\eta_{inc,gap} = 1.1$, which is the value used in Eq. (S3.2). The focusing process is sensitive to the launching of the correct wavefronts at the in-coupling grating, whereas the de-focusing process is not. The asymmetry in values of $\eta_{inc,gap}$ and $\eta_{out,gap}$, is necessary to account for the imperfect focussing of waves in the slab by the curved gratings. The focussed field at the gap does not perfectly match the field distribution of the gap mode. However, on de-focussing, the plasmonic gap mode spreads out into waves of the slab that are all scattered by the output grating. We expect that a perfectly implemented coupling grating could ensure focusing and de-focusing with

the same efficiency. The values for $\eta_{meas}(L = 0)$ at each measured gap width are listed in the caption of Figure S4.

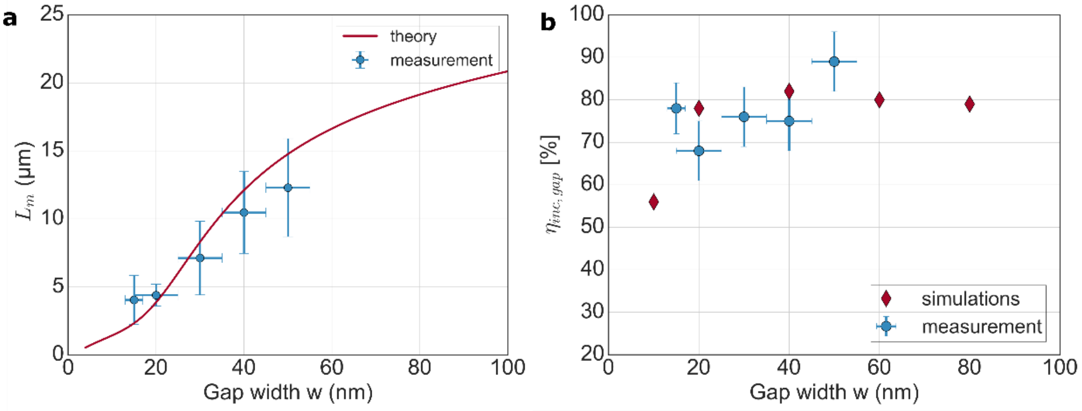


**Figure S5 |** **a** Extracted propagation length from the cut-back method measurements shown in Figure S4 for various gap widths, compared to theoretical eigenmode-solver calculations. **b** Extracted gap in-/out-coupling efficiencies compared to 3D FDTD simulations of the entire system at a wavelength of 1550 nm. Data points in **a** and **b** are the mean and error bars the standard deviation.

Figure S4a shows the propagation length for various gap widths, defined as the length at which the incident intensity of a propagating surface plasmon mode drops to $e^{-1}$ of its initial value. Figure S4b compares the measured (extracted from Figure S4) and calculated coupling efficiencies. The latter has been determined from 3D FDTD simulations[9]. The result demonstrates a good agreement between experiment and theory, which underpins the good performance of this nanofocusing platform with a gap coupling efficiency of $\eta_{inc,gap} \approx 80$ %. Based on this result, the out-coupling efficiency of erbium luminescence (out of the gap and guided to the grating) has been conservatively estimated to be 80 %. This is likely to be closer to 90 %, consistent with simulations; however, this cannot be verified experimentally.

## 4. Luminescence background

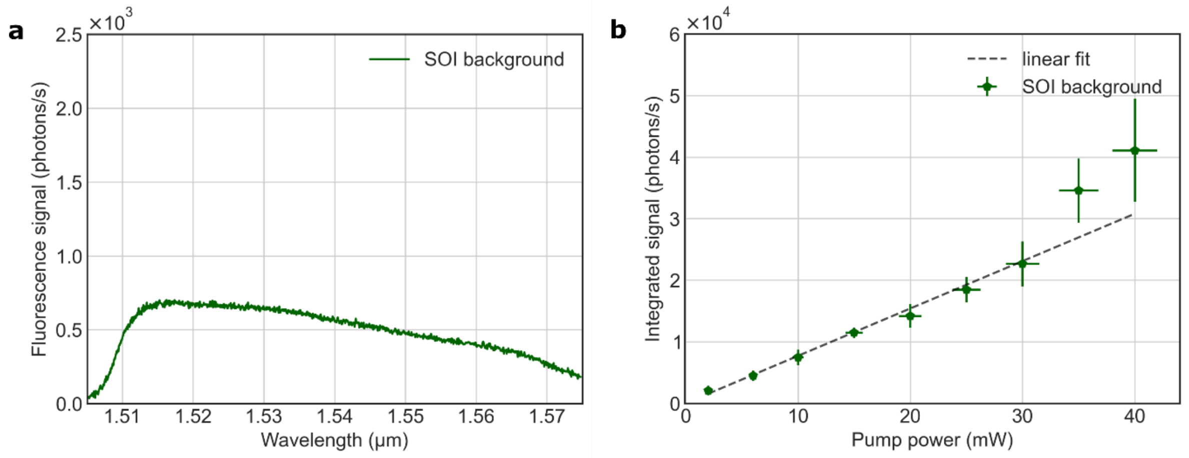


**Figure S6| Background luminescence from a non $Er^{3+}$-ion containing Si reference sample. a**, Luminescence spectra from a no ion containing reference sample measured at 40 mW pump power (980 nm, CW). The sample was annealed analogously to the actual devices used in the main manuscript. **b**, Power dependent measurement showing the integrated signal from **(a)** at different pump powers. Data points are the mean and the error bars are the standard deviation. The dashed lines indicate the theory fits to each curve. Linear fitting was performed to estimate the Si background subtracted from the measurement in the manuscript.

To account for background luminescence from the sample, reference measurements were performed on a plain SOI sample without erbium ions. The sample was coated with 25 nm $SiO_2$, identical to the ion containing sample. The "SOI background" reference sample spectra is shown in Figure S6a. This sample was annealed under the same conditions as the ion containing sample described in the main manuscript but does not possess any patterns or fabricated structures. To provide a conservative overall estimate of the Purcell enhancement factor, $\langle F_p \rangle$, we assumed a linear power dependence of the SOI background (Figure S6b). The slight non-linear trend may arise from a nonlinear behaviour of the photodetector for the very low count rates of the non-ion containing samples. For the estimate of $I_{i,sat}$ and eventually $\langle F_p \rangle$ in the main manuscript, the linear fitted power dependence of the background was subtracted from the measured luminescence power dependencies of the ion-containing samples. For the Wg devices, the background signal was multiplied by the fraction of the illuminated sample area which is not covered by the Au taper cladding (e.g.,$12\% * I_{background}$). The background subtraction is used for the curves in the luminescence power dependencies shown in Figure 4c.

## 5. Rate equation model and the Purcell factor $F_P$ in saturation regime

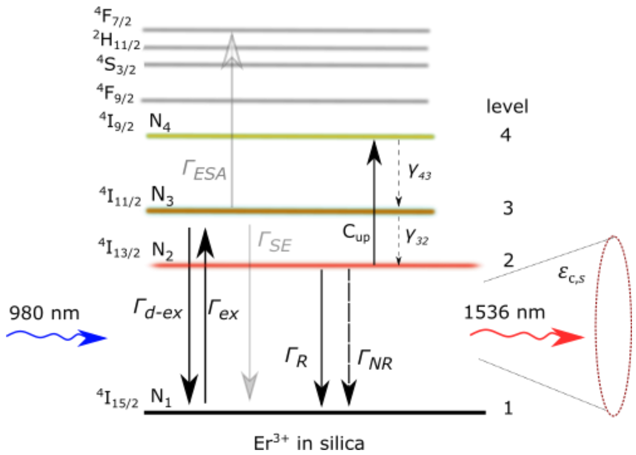


**Figure S7| Schematic energy diagram illustrating the relevant $Er^{3+}$-ion energy level,** (with population numbers $N_1$, $N_2$, $N_3$, $N_4$) and corresponding transitions probed in the experiment. The 980 nm pump and the 1536 nm emission signal (peak wavelength) are shown in blue and red, respectively. $\Gamma_{ex}$ and $\Gamma_{d-ex}$ are the excitation and de-excitation rates between $N_3$ and $N_1$. $\gamma_{43}$ and $\gamma_{32}$ are the transition rates from level 4 to level 3 and 3 to 2, respectively. $\Gamma_R$ and $\Gamma_{NR}$ describe the radiative and non-radiative transitions from level 2 to 1 while $C_{up}$ is the cooperative up-conversion coefficient. $\varepsilon_{c,s}$ is the collection efficiency of light emitted by the transition from level 2 to level 1. $\Gamma_{ESA}$ is the excited state absorption (ESA) rate which however can be neglected as explained in the text. $\Gamma_{SE}$ is the spontaneous emission rate which can also be neglected due to a small branching ratio as explained in the text. Hence merely the colored energy levels are relevant for the rate equations in this particular experiment.

Figure S7 shows the energy levels of $Er^{3+}$ with the relevant transitions studied in the experiment. The experimental settings are as follows: erbium is implanted in a pure $SiO_2$ host matrix and the pump wavelength is 980 nm. No sensitizers (such $Yb^{3+}$) or co-dopants (Al-P, Ge, Eu, or fluorides) or low-phonon energy host matrixes (such as $Y_2O_3$) which increase the state lifetimes have been employed. Plasmonic metal structures are placed on top of the ion implanted layer. Although plasmonics can be used in this context to enhance the erbium ion excitation[10] the structure used here is neither an antenna nor a cavity and does not contribute to a significant excitation enhancement as shown in Figure S1 a2. Importantly, in the pump saturation regime, the emission intensity is independent of the pump power and hence the excitation. The values provided for the radiative Purcell enhancement can be based on the emission intensities in saturation and are thus independent of any plasmonic excitation enhancing effects.

The excitation and recombination dynamics of erbium can involve excited state absorption (ESA) and energy transfer effects such a cooperative up-conversion (CUP)[11]. The relevance for an accurate description of carrier dynamics of each of those effects, depends on the pump wavelength, the host matrix as well as the ion concentration and the pump power. In this study, ESA due to the 980 nm pump can be neglected, both from the $I_{11/2} \rightarrow F_{7/2}$ level as well as from the $I_{13/2}$ level. For the latter no possible energy transition exists while various studies showed that ESA from the $I_{11/2} \rightarrow I_{9/2}$ transition can be neglected[12–15]. This is due to the high phonon energy of the pure silica host matrix leading to a rapid de-population of the $I_{11/2}$ level ( ~5 µs)[15–19]. Note that studies which include ESA for a 980 nm

pump from the $I_{11/2}$ level[20,21] are often based on non-pure $SiO_2$ (e.g. Al/P-silica) fibers which show a longer $I_{11/2}$ level lifetime. Moreover, spontaneous emission from the $I_{11/2}$ level can be neglected due to a branching ratio of $>10^4$ [16].

Due to the doping concentration used in the experiment we take CUP into account. During CUP two ions in the $I_{13/2}$ level interact leading to a de-excitation of one of the ions and an excitation to the $I_{9/2}$ level of the other. Note however that the lifetime of the $I_{9/2}$ state is on the order of ns[15] and the $I_{11/2}$ on the order of µs, leading to a rapid de-population of the $I_{9/2}$ state back to the $I_{13/2}$ state. Based on these considerations and the literature[11,17], we derive an effective three level rate equation, respectively while taking rapid up-conversion as an additional non radiative transition channel into account. This is outlined in the following.

The measured luminescence signal $S_S$ of an ion coupled to an optical mode (either photonic or plasmonic) can be expressed by the radiative de-population rate $\Gamma_R$ of energy level 2 with population $N_2$ (c.f. Fig. S7) and the emission collection efficiency $\varepsilon_{c,s}$:

$$S_S = \varepsilon_{c,s} N_2 \Gamma_R \, . \qquad \text{(S5.1)}$$

As the population $N_2$ and its radiative de-population rate $\Gamma_R$ are not directly accessible in this experiment, we will use a rate equation model to find an experimentally accessible expression. The excitation and recombination dynamics illustrated in Figure S7 can be described by Einstein's rate equations[22]:

$$\dot{N}_2 = -\Gamma_R N_2 - \Gamma_{NR} N_2 - 2\, C_{up} {N_2}^2 + \gamma_{32} N_3 \qquad \text{(S5.2)}$$

$$\dot{N}_3 = (N_1 - N_3)\, \Gamma_{ex} - \gamma_{32} N_3 + \gamma_{43} N_4 \qquad \text{(S5.3)}$$

$$\dot{N}_4 = C_{up} {N_2}^2 - \gamma_{43} N_4 \qquad \text{(S5.4)}$$

$$N = N_1 + N_2 + N_3 + N_4, \qquad \text{(S5.5)}$$

where $N$ is the total population number of the system, $\gamma_{43}$ and $\gamma_{32}$ are the transition rates from level 4 to level 3 and 3 to 2, respectively with populations numbers $N_4$, $N_3$ and $N_2$, $\Gamma_{NR}$ is the non-radiative recombination rate, $C_{up}$ is the up-conversion coefficient and $\Gamma_{ex}$ is the excitation rate of carriers from the ground state to level 3. Under steady state illumination, we assume that the excitation rate is proportional to optical pump power, $\Gamma_{ex} \propto P$ and assume a steady state solution: $\dot{N}_2 = \dot{N}_3 = \dot{N}_4 = 0$. Due to the rapid de-population of the third (~µs lifetime) and fourth levels (~ns lifetime) described above, $N_3 \approx 0$ and $N_4 \approx 0 \Rightarrow N_1 \approx N - N_2$[17,23]. Adding equations S5.2 – S5.4 now yields:

$$N_2 \Gamma_R = \underbrace{\frac{N \Gamma_{ex} \Gamma_R}{[\Gamma_{ex} + \Gamma_R + \Gamma_{NR}]}}_{a(\Gamma_{ex})} - \underbrace{\frac{C_{up} {N_2}^2 \Gamma_R}{[\Gamma_{ex} + \Gamma_R + \Gamma_{NR}]}}_{b(\Gamma_{ex})} \qquad \text{(S5.6)}$$

Where $a(\Gamma_{ex})$ is the expression for low ion density limit (i.e. for $C_{up} = 0$) describing the transition rate dynamics in a three level emitter system[24] minus a correction term $b(\Gamma_{ex})$ to account for potential up-conversion. We estimate this correction with respect to the conventional three-level model in the signal saturation regime:

$$\frac{b(\Gamma_{ex})}{a(\Gamma_{ex})} = \frac{C_{up}{N_2}^2}{\Gamma_{ex}N} \leq \frac{C_{up}N}{\Gamma_{ex}} \rightarrow 0 \text{ for } \Gamma_{ex} \gg C_{up}N \tag{S5.7}$$

Here, the conventional as well as the up-conversion corrected model yield the same result. One may use the conventional expression $a(\Gamma_{ex})$ which neglects CUP when working in saturation if the conditions i) $\gamma_{32} \gg \Gamma_{ex}$ and ii) $\Gamma_{ex} \gg C_{up}N$ are fulfilled, whereas $\gamma_{32} \sim 2 \cdot 10^6 s^{-1}$ (i.e. ~5 μs[15–19] ) and $C_{up}N \sim 1 \times 10^3$ $s^{-1}$. Here we used $C_{up} \sim 10^{-24}$ $m^3s^{-1}$ [22] and the total number of ions $N \sim 10^{27}$ extracted from implantation calculations provided in S3.

For the non-enhanced Gr-to-Gr control sample near saturation the measured recombination rate is on the order of the excitation rate $\Gamma_{ex,Gr} \sim 10^3$ $s^{-1}$ which is close to violating condition (ii). Note however, that up-conversion diminishes the luminescence signal, hence this estimate overestimates the signal from the Gr control sample ($S_{Gr}^{40mW}$), leading to a valid lower bound of the enhancement factor in the manuscript, as EF ∝ $\frac{S_{Gap}^{40mW}(w)}{S_{Gr}^{40mW}}$.

For the plasmonic gap waveguide sample the excitation rate is $\Gamma_{gap,ex} \sim 10^6$ $s^{-1}$, as shown in Figure 3f. While this is in bounds of condition (ii), it may violate condition (i) if transition 3 → 2 remains non-Purcell enhanced.

With this in mind, we re-consider the rate equation model (S5.2 – S5.5) without relying on condition (i). Considering the premise of a non-negligible population of the third energy level due to CUP, i.e. $N_3 \neq 0$ but with $N_4 \approx 0$ and adding Eq. (S5.3) to (S5.4) yields:

$$N_3 = \frac{C_{up}{N_2}^2 + \Gamma_{ex}N_1}{(\gamma_{32} + \Gamma_{ex})}. \tag{S5.8}$$

Inserting into Eq. (S5.2) and re-writing yields:

$$\Gamma N_2 + 2\, C_{up}{N_2}^2 = \frac{\gamma_{32}}{(\gamma_{32} + \Gamma_{ex})}\left(C_{up}{N_2}^2 + \Gamma_{ex}N_1\right), \tag{S5.9}$$

where $\Gamma = \Gamma_R + \Gamma_{NR}$. Using

$$N_1 = N - N_2 - N_3, \tag{S5.10}$$

and substituting for $N_3$ based on Eq. (S5.8) gives a recurring relationship in $N_1$:

$$\Gamma N_2 + \left(2 - \frac{\gamma_{32}}{(\gamma_{32} + \Gamma_{ex})}\right) C_{up}{N_2}^2 = \Gamma_{ex}\frac{\gamma_{32}}{(\gamma_{32} + \Gamma_{ex})}\left(N - N_2 - \frac{\left(C_{up}{N_2}^2 + \Gamma_{ex}N_1\right)}{(\gamma_{32} + \Gamma_{ex})}\right), \tag{S5.11}$$

as shown down below for the first few terms, by alternating insertion of Eq. (S5.10) and Eq. (S5.8):

$$\Gamma N_2 + \left(2 - \frac{\gamma_{32}}{(\gamma_{32}+\Gamma_{ex})}\right) C_{up}{N_2}^2 = \Gamma_{ex}\frac{\gamma_{32}}{(\gamma_{32}+\Gamma_{ex})}\left[N - N_2 - \frac{C_{up}{N_2}^2}{(\gamma_{32}+\Gamma_{ex})} - \frac{\Gamma_{ex}}{(\gamma_{32}+\Gamma_{ex})}\left(N - N_2 - \frac{C_{up}{N_2}^2}{(\gamma_{32}+\Gamma_{ex})}\right) + \frac{\Gamma_{ex}^2}{(\gamma_{32}+\Gamma_{ex})^2}\left(N - N_2 - \frac{C_{up}{N_2}^2}{(\gamma_{32}+\Gamma_{ex})}\right) - \cdots\right]. \quad \text{(S5.12)}$$

We can identify a geometric series from Eq. (S5.12):

$$\Gamma N_2 + \left(2 - \frac{\gamma_{32}}{(\gamma_{32}+\Gamma_{ex})}\right) C_{up}{N_2}^2 = \Gamma_{ex}\frac{\gamma_{32}}{(\gamma_{32}+\Gamma_{ex})}\left(1 - \frac{\Gamma_{ex}}{(\gamma_{32}+\Gamma_{ex})} + \frac{\Gamma_{ex}^2}{(\gamma_{32}+\Gamma_{ex})^2} - \cdots\right)$$

$$\left(N - N_2 - \frac{C_{up}{N_2}^2}{(\gamma_{32}+\Gamma_{ex})}\right). \quad \text{(S5.13)}$$

The geometric series can be written as,

$$1 - \frac{\Gamma_{ex}}{(\gamma_{32}+\Gamma_{ex})} + \frac{\Gamma_{ex}^2}{(\gamma_{32}+\Gamma_{ex})^2} - \cdots = \frac{1}{1+\frac{\Gamma_{ex}}{(\gamma_{32}+\Gamma_{ex})}} = \frac{(\gamma_{32}+\Gamma_{ex})}{\gamma_{32}+2\Gamma_{ex}}. \quad \text{(S5.14)}$$

Substituting Eq. (S3.14) back into Eq. (S3.11) yields:

$$\Gamma N_2 + \left(2 - \frac{\gamma_{32}}{(\gamma_{32}+\Gamma_{ex})}\right) C_{up}{N_2}^2 = \Gamma_{ex}\frac{\gamma_{32}}{(\gamma_{32}+2\Gamma_{ex})}\left(N - N_2 - \frac{C_{up}{N_2}^2}{(\gamma_{32}+\Gamma_{ex})}\right) \quad \text{(S5.15)}$$

From here, we further simplify the expression

$$N_2\left(\Gamma + \Gamma_{ex}\frac{\gamma_{32}}{(\gamma_{32}+2\Gamma_{ex})}\right) + \left(2 - \frac{1}{(\gamma_{32}+\Gamma_{ex})}\left(\gamma_{32} + \Gamma_{ex}\frac{\gamma_{32}}{(\gamma_{32}+2\Gamma_{ex})}\right)\right) C_{up}{N_2}^2 = \Gamma_{ex}\frac{\gamma_{32}}{(\gamma_{32}+2\Gamma_{ex})}N \quad \text{(S5.16)}$$

and identify $\tilde{\Gamma}_{ex} = \Gamma_{ex}\frac{\gamma_{32}}{(\gamma_{32}+2\Gamma_{ex})}$ as the altered excitation rate taking CUP into consideration:

$$(\Gamma + \tilde{\Gamma}_{ex})N_2 + \left(2 - \frac{(\gamma_{32}+\tilde{\Gamma}_{ex})}{(\gamma_{32}+\Gamma_{ex})}\right) C_{up}{N_2}^2 = N\tilde{\Gamma}_{ex}\,. \quad \text{(S5.17)}$$

Providing a high depopulation rate from level 3 to 2, $\gamma_{32} \gg \Gamma_{ex}$, we retrieve Eq. (S5.6):

$$(\Gamma + \Gamma_{ex})N_2 + \ C_{up}{N_2}^2 - \Gamma_{ex}N = 0.$$

This expression is valid for low pump rates ($\gamma_{32} \gg \Gamma_{ex}$), as discussed above. However, without restriction of $\gamma_{32} \gg \Gamma_{ex}$, we can solve the quadratic equation (S5.17) with $\alpha = 2 - \frac{(\gamma_{32}+\tilde{\Gamma}_{ex})}{(\gamma_{32}+\Gamma_{ex})}$

$$(\Gamma + \tilde{\Gamma}_{ex})N_2 + \alpha C_{up}{N_2}^2 = N\tilde{\Gamma}_{ex}. \quad \text{(S5.18)}$$

With the physical, positive solution:

$$N_2 = -\frac{1}{2\alpha C_{up}}(\Gamma + \tilde{\Gamma}_{ex}) + \frac{1}{2\alpha C_{up}}(\Gamma + \tilde{\Gamma}_{ex})\sqrt{1 + 4\alpha\frac{C_{up}N}{\Gamma'_{ex}}\frac{{\tilde{\Gamma}_{ex}}^2}{(\Gamma_T+\tilde{\Gamma}_{ex})^2}}\,. \quad \text{(S5.19)}$$

Providing $C_{up}N \ll \tilde{\Gamma}_{ex}$ (condition (ii) discussed above), we can approximate Eq. (S5.19) to:

$$N_2 \approx \frac{1}{2\alpha C_{up}}\left(\Gamma + \tilde{\Gamma}_{ex}\right)\left(-1 + 1 + 2\alpha \frac{C_{up}N}{\tilde{\Gamma}_{ex}} \frac{\tilde{\Gamma}_{ex}{}^2}{(\Gamma+\tilde{\Gamma}_{ex})^2}\right) = N\frac{\tilde{\Gamma}_{ex}}{(\Gamma+\tilde{\Gamma}_{ex})} \quad \text{(S5.20)}$$

The result provided in form of Eq. (S5.20) demonstrates that the functional form of Eq. (S5.6) is maintained in presence of CUP while the excitation rate now can be expressed as $\tilde{\Gamma}_{ex} = \Gamma_{ex}\frac{\gamma_{32}}{(\gamma_{32}+2\Gamma_{ex})}$. Note that this result allows to use a three-level model description with a strictly monotonically increasing signal in dependence on pump power, in analogy to the conventional three-level model[24], and hence validates the relation $Fp > EF$.

Substitution of Eq. (S5.20) into Eq. (S5.1) yields:

$$S_s = \frac{\varepsilon_{c,s} N \tilde{\Gamma}_{ex} \eta_{q,s}}{\left[1+\frac{\tilde{\Gamma}_{ex}}{\Gamma}\right]}, \quad \text{(S5.21)}$$

where $\Gamma = \Gamma_R + \Gamma_{NR}$ is the total de-population rate, which takes the radiative and non-radiative recombination into account. Accordingly, the internal quantum efficiency $\eta_{q,s} = \Gamma_R/\Gamma$ is identified.

For our Gr-Gr configuration control sample, the measured signal, $S_{Gr}$, can be expressed in analogue to Eq. (S5.20):

$$S_{Gr} = \varepsilon_{c,Gr}\tilde{N}_{Gr}\Gamma_R \frac{\tilde{\Gamma}_{ex,Gr}/\Gamma}{\left[1+\frac{\tilde{\Gamma}_{ex,Gr}}{\Gamma}\right]}, \quad \text{(S5.22)}$$

where the total population number $\tilde{N}_{Gr}$ is directly proportional with a constant, $a$, to the number of illuminated ions $\tilde{N}_{Gr} = aN_{Gr}$ in the Gr-Gr configuration. $\varepsilon_{c,Gr}$ is the photon coupling efficiency to the detector, $\tilde{\Gamma}_{ex,Gr}$ the excitation rate from the ground level, and $\eta_{q,Gr}$ the internal quantum efficiency as defined for the general case in Eq. (S5.21).

Let us now take the Purcell effect into account to describe the accelerated emission of the hybrid plasmonic gap waveguide devices. The Purcell enhancement factor $F_p$ acts only upon the radiative process, with recombination rate $\Gamma_R$, given that the non-radiative rate $\Gamma_{NR}$ is due to internal processes of the atomic system and is not affected by altering the electromagnetic density of states. However, non-radiative transitions can be enhanced in plasmonic structures due to e.g. metal quenching, which we take into account by a non-radiative loss factor $F_{NR}$. Hence, a modified total de-population rate $\Gamma'$ and modified internal quantum efficiency $\eta_{q,Gap}$ are defined:

$$\Gamma \rightarrow \Gamma' = F_p\Gamma_R + F_{NR}\Gamma_{NR}, \quad \text{(S5.23)}$$

$$\eta_{q,s} \rightarrow \eta_{q,Gap} = \frac{F_p\Gamma_R}{[F_p\Gamma_R + F_{NR}\Gamma_{NR}]}. \quad \text{(S5.24)}$$

Analogously to Eq. (S5.22), we can write the measured signal of $Er^{3+}$-ion emitters in the hybrid plasmonic gap waveguide devices using the substitutions ($\Gamma'$ and $\eta_{q,Gap}$),

$$S_{Gap} = \varepsilon_{c,Gap} \widetilde{N}_{Gap} F_p \Gamma_R \frac{\tilde{\Gamma}_{ex,Gap}/\Gamma'}{\left[1+\frac{\tilde{\Gamma}_{ex,Gap}}{\Gamma'}\right]}, \qquad \text{(S5.25)}$$

where the total population $\widetilde{N}_{Gap}$ is directly proportional to the number of illuminated gap ions $\widetilde{N}_{Gap} = aN_{Gap}$ in the experiment. $\varepsilon_{c,Gap}$ is the photon coupling efficiency to the detector, $\tilde{\Gamma}_{ex,Gap}$ the excitation rate and $\eta_{q,Gap}$ the internal quantum efficiency as defined for the general case in Eq. (S5.21).

In the high pump power case, $P \propto \tilde{\Gamma}_{ex,Gr} \gg \Gamma = \Gamma_R + \Gamma_{NR}$, leaving us with:

$$S_{Gr} \approx \varepsilon_{c,Gr} \widetilde{N}_{Gr} \Gamma_R, \qquad \text{(S5.26)}$$

for the Gr-Gr device and $P \propto \tilde{\Gamma}_{ex,Gap} \gg \Gamma' = F_p \Gamma_R + F_{NR} \Gamma_{NR}$:

$$S_{Gap} \approx \varepsilon_{c,Gap} F_p \widetilde{N}_{Gap} \Gamma_R, \qquad \text{(S5.27)}$$

for the gap plasmon waveguide. The ratio of these saturated signals yields an expression for the Purcell factor $F_p$:

$$\frac{S_{Gap}}{S_{Gr}} = \frac{\varepsilon_{c,Gap}}{\varepsilon_{c,Gr}} \frac{N_{Gap}}{N_{Gr}} F_p\ . \qquad \text{(S5.28)}$$

While expression S5.28 allows us to estimate the Purcell factor in the saturation regime, we can also provide a lower estimate for $F_p$ directly from the measurements in Figure 4c, given by:

$$\boxed{F_p \geq \frac{S_{Gap}(w,\Gamma_{ex})}{S_{Gr}(w,\Gamma_{ex})} \frac{A_{Gr}}{L \cdot \mathrm{w}} \frac{\varepsilon_{c,Gr}(w)}{\varepsilon_{c,Gap}(w)} \equiv \mathrm{EF}} \qquad \text{(S5.29)}$$

, where we now defined the luminescence enhancement factor EF and replaced $N_{Gr}/N_{Gap}$, with the ratio of illuminated ions in the Gr and the gap configuration: $A_{Gr}/(L \cdot w)$. The relation is a valid lower estimate for $F_p$ even at powers below the saturation regime as $\frac{S_{Gap}(w,\Gamma_{ex})}{S_{Gr}(w,\Gamma_{ex})}$ is strictly monotonically increasing with pump power (with $\Gamma_{ex} \propto P$). This is due to the fact that luminescence from the gap waveguide is strongly increased while the total emission rate (i.e. faster depletion of $\mathrm{N}_2$) is larger for the gap waveguide than for the Gr sample, leading to higher saturation powers and larger saturation of luminescence values for gap waveguides compared to the Gr-to-Gr device. Note, that field enhancement due to the plasmonic structure at the ion position, i.e. 1 nm below the gap, is merely a factor of about two on average as shown in Figure S1 a2 and can be excluded as the origin of the several hundred fold emission enhancement.

## 6. Gaussian saturation model and Purcell enhancement results

Although we use Eq. S5.29 to provide an estimate for the luminescence enhancement EF, we are also providing a fitting model based on the conventional three level model (i.e. $C_{up}$=0) below in order to show that the simple model provides a good description of the measured data. This hints to the fact that cooperative up-conversion effects are potentially small. For this, we adapt the saturation model from Section 4 to the Gaussian excitation beam distribution used in the experiment.

Here, we use the fact that the excitation rate $\Gamma_{ex}$ is linearly proportional to the pump beam's intensity, $\mathcal{I}(x,y)$, i.e., $\Gamma_{ex} = \gamma_{\text{ex}}\mathcal{I}(x,y)$. Following Eq. (S5.21) we can write the output intensity from the three-level model for the Gr-Gr case as,

$$I = c\varepsilon_C \Gamma_R \frac{\mathcal{I}(x,y)/\mathcal{I}_{sat}}{1+\mathcal{I}(x,y)/\mathcal{I}_{sat}}, \tag{S6.1}$$

where $\mathcal{I}_{sat} = \Gamma/\gamma_{ex}$ is the saturation intensity, $c$ is the erbium ion areal density, $\varepsilon_C$ is the coupling efficiency of ion emission to the detector, and $\Gamma_R$ is the recombination rate of erbium ions. For a Gaussian beam, $\mathcal{I}(x,y) = ae^{-2r^2/r_0^2}$, we find a relationship with excitation power, $P = \pi r_0^2 a/2$, so that,

$$I = c\varepsilon_C \Gamma_R \frac{e^{-2r^2/r_0^2}P/P_{sat}}{1+e^{-2r^2/r_0^2}P/P_{sat}} \tag{S6.2}$$

where, $\mathcal{I}_{sat}\pi r_0^2/2 = P_{sat}$, is the saturation power. To determine the total collected signal, $S(P)$, that would be measured in an experiment, we integrate over all excited ions,

$$S(P) = c\varepsilon_C \Gamma_R \int_0^{2\pi} d\phi \int_0^{\infty} r dr \frac{e^{-2r^2/r_0^2}P/P_{sat}}{1+e^{-2r^2/r_0^2}P/P_{sat}}. \tag{S6.3}$$

Here we have assumed that the collection efficiency, areal density, and emission rate of the excited ions are uniform across the illumination area. To simplify, we distinguish the saturation function from the saturation signal by substituting for a normalised integration variable: $\rho = \sqrt{2}r/r_0$, to find:

$$S(\mathrm{P}) = \frac{1}{2} r_0^2 c\varepsilon_C \Gamma_R \int_0^{2\pi} d\phi \int_0^{\infty} \rho d\rho \frac{e^{-\rho^2}P/P_{sat}}{1+e^{-\rho^2}P/P_{sat}} \tag{S6.4}$$

We use this saturation model to evaluate the saturation of ions in the gap of the plasmonic waveguide (Wg device) and the $SiO_2$ on the SOI slab waveguide surface (Gr device).

**Saturation Model for the Gr-Gr Control Sample:**

For the Gr-Gr control sample, we need only to evaluate the integral, to find,

$$S_{Gr}(P) = \frac{1}{2} A_{Gr} c \varepsilon_{C,Gr} \Gamma_R \ln\left(1 + P/P_{Gr,sat}\right) = S_{Gr,sat} \ln\left(1 + P/P_{Gr,sat}\right) \tag{S6.5}$$

where $A_{Gr} = \pi r_0^2$, is the area of the Gaussian beam and $\varepsilon_{C,Gr}$ is the collection efficiency of erbium emission for the Gr-Gr sample. This equation can be fit to the control sample data using two free parameters, $S_{Gr,sat}$ and $P_{Gr,sat}$. For the computation of the Purcell factor, we will want to find a value for $S_{Gr,sat} = A_{Gr} c \varepsilon_{C,Gr} \Gamma_R / 2$, from the fitted experimental data.

**Saturation Model for the Gap Plasmon Samples:**

For the gap waveguide samples, erbium emission originates from the gap and taper regions, which we treat separately. For the taper region, we assume that the erbium emission is related to that of the Gr-Gr control sample but limited by the physical masking of the erbium by the metal structure that defines the taper. For a taper opening angle $\alpha$ as defined in the main text, we find,

$$S_{taper}(P) = \alpha r_0^2 c \varepsilon_{C,Gr} \Gamma_R \int_0^\infty \rho d\rho \frac{e^{-\rho^2} P/P_{Gr,sat}}{1 + e^{-\rho^2} P/P_{Gr,sat}} = \frac{\alpha}{\pi} S_{Gr}(P) \tag{S6.6}$$

For the erbium ions in the gap, we assume a near uniform excitation profile ($e^{-2r^2/r_0^2} \approx 1$) to produce the conventional saturation response function:

$$S_{Gap}(P) \approx c \varepsilon_C F_p \Gamma_R \frac{P/P_{Gap,sat}}{1 + P/P_{Gap,sat}} \int_{-w/2}^{w/2} dy \int_{-L/2}^{L/2} dx = A_{gap} c \varepsilon_{C,Gap} \Gamma_R F_p \frac{P/P_{Gap,sat}}{1 + P/P_{Gap,sat}}, \tag{S6.7}$$

where $A_{gap} = wL$ is the area of the gap region, $\varepsilon_{C,Gap}$ is the collection efficiency of ions from the gap region, and $F_P$ is the Purcell factor. The total saturation response of the gap waveguide sample is now described by $S_h(P) = S_{Gap}(P) + S_{taper}(P)$, such that

$$S_h(P) \approx S_{Gap,sat} \frac{P/P_{Gap,sat}}{1 + P/P_{Gap,sat}} + \frac{\alpha}{\pi} S_{Gr,sat} \ln\left(1 + P/P_{Gr,sat}\right) \tag{S6.8}$$

where $S_{Gap,sat} = A_{gap} c \varepsilon_{C,Gap} \Gamma_R F_p$ and $S_{Gr,sat} = A_0 c \varepsilon_{C,Gr} \Gamma_R / 2$. Here again, only two fitting parameters are used, $S_{Gap,sat}$ and $P_{Gap,sat}$, as $S_{Gr,sat}$ and $P_{Gr,sat}$ are taken from the model fitting (Eq. (S6.5)) to the Gr-Gr sample data. The parameters extracted from fitting Eq. (S6.5) and Eq. (S6.8) to the measured integrated luminescence as a function of pump power and for different gap widths, shown in Figure 4c of the main manuscript, are listed in Table S1.

## Evaluating the Purcell Factor from Saturation Curves

**Table S1| Fit parameters and reduced $\chi^2_{red}$,** extracted from least square optimization of the fit functions for $S_i(P)$ with $i = \{Gr, Gap\}$, describing the power dependency of the integrated measured emission spectra in Gr-to-Gr and Wg configuration for different gap widths $w$.

| Gap width $w$ | $S_{i,sat}$ (photons/s) | $P_{i,sat}$ (mW) | $\chi^2_{red}$ |
|---|---|---|---|
| 10 nm | $(80 \pm 11) * 10^4$ | $72 \pm 14$ | 1.31 |
| 17 nm | $(59 \pm 16) * 10^4$ | $91 \pm 30$ | 0.45 |
| 30 nm | $(47 \pm 14) * 10^4$ | $113 \pm 44$ | 1.03 |
| 60 nm | $(22 \pm 4) * 10^4$ | $58 \pm 15$ | 3.21 |
| Gr-Gr | $(10 \pm 1) * 10^4$ | $23 \pm 4$ | 1.01 |

**Table S2| Simulated radiative Purcell factor $F^c_{p,1\,nm}$ averaged across the lateral dipole positions of the gap waveguide at 1 nm below the interlayer oxide/gap interface**. The values are listed for various gap widths $w$. $\varepsilon_{c,Gr}$ and $\varepsilon_{c,Gap}$ are the collection efficiency via the gratings and the fundamental gap mode, respectively. $\frac{A_{Gr}}{L \cdot w}$ is the ratio between the illumination area in the Gr-to-Gr configuration, $A_{Gr}$ and the area of the waveguide gap $A_{Gap} = w\,L$ of length $L$ and width $w$. All values are for an emission wavelength of λ = 1.536 µm.

| Gap width $w$ | $\varepsilon_{c,Gr}/\varepsilon_{c,Gap}$ | $\frac{A_{Gr}}{w\,L}$ | $\frac{S_{Gap}(w, 40\,mW)}{S_{Gr}(w, 40\,mW)}$ | EF | $F^c_{p,1\,nm}$ |
|---|---|---|---|---|---|
| 10 nm | 0.23 | $317 \pm 100$ | $4.63 \pm 0.19$ | $338 \pm 109$ | $596 \pm 182$ |
| 17 nm | 0.23 | $187 \pm 38$ | $2.24 \pm 0.23$ | $96 \pm 22$ | $224 \pm 57$ |
| 30 nm | 0.23 | $106 \pm 15$ | $1.27 \pm 0.07$ | $31 \pm 5$ | $103 \pm 54$ |
| 60 nm | 0.23 | $53 \pm 6$ | $0.58 \pm 0.05$ | $7 \pm 1$ | $62 \pm 50$ |

Table S2 lists the values used to calculate the emission enhancement factor per ion EF (Figure 4f of the main manuscript) which is a lower estimate for the Purcell factor $F_p$. Note that the intensity of the pump field which excites the ions drops exponentially to each side of the gap as shown by simulations in Figure S1 a3 (logarithmic scale). Hence, the number of illuminated ions can be estimated based on the gap width $w$.

Table S2 also lists the computed Purcell enhancement $F^c_{p,1\,nm}$ extracted from measurement and simulations for a dipole with a distance of Δd = 1 nm below the gap interface. In simulations, we average the purely radiative Purcell factor $F_p$ of a single dipole emitter over different lateral positions across the gap for one specific implantation depth Δd (Figure S8). We average these values across the

individual position and for each gap width to extract the average computed Purcell factor $F_p^c(w, \Delta d)$ (Fig. 3f and dashed lines Figure S8). The simulations are performed for an electric dipole oriented perpendicular to the gap waveguide direction (TE) positioned at a distance of Δd below the Au/$SiO_2$

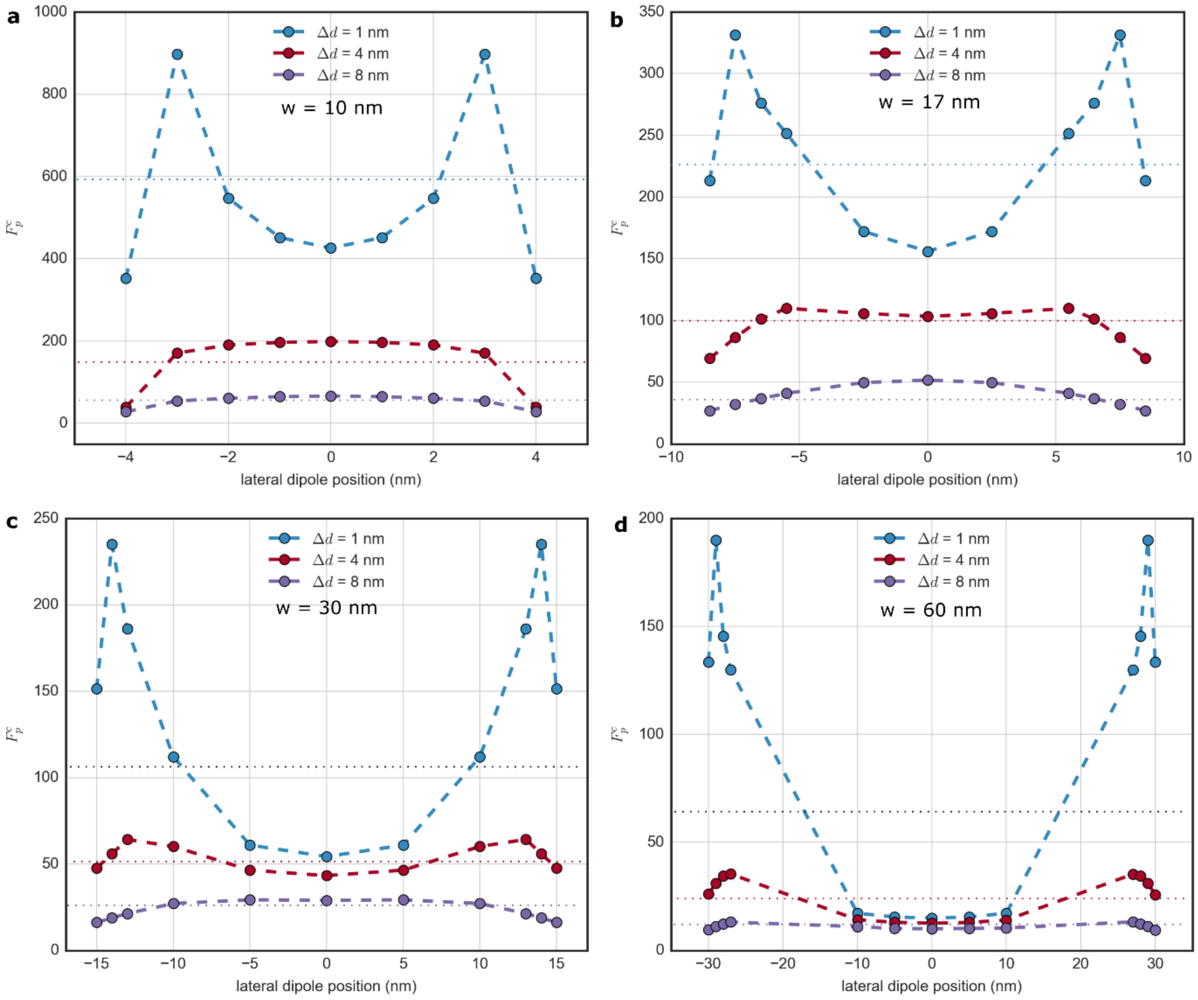


**Figure S8| FDTD simulations showing the radiative Purcell enhancement** $F_p$ for a perpendicular to the MIM gap aligned electrical dipole at different distances, *Δd* = {1 nm, 4 nm, 8 nm} below the $SiO_2$/Au gap interface for various lateral positions (centre gap position at 0 nm). Radiative Purcell enhancement for MIM gap widths of **a** 10 nm, **b** 17 nm, **c** 30 nm and **d** 60 nm all for a wavelength of 1.536 µm and a 50 nm thick Au gap layer. These values were averaged for each gap width to provide $F_p^c(w, \Delta d)$ highlighted by horizontal dashed lines.

interface. Table S2 list the result for *Δd* = 1 nm. The simulation results of the individual Purcell factor, $F_p$, are shown exemplarily in Figure S6 for the *Δd* = 1 nm, *Δd* = 4 nm and *Δd* = 8 nm as described in the main text for 1 nm and 4 nm (Fig. 3a). The simulated $F_p^c$ neglects the $F_p$ values for dipoles with a distance of < 1 nm from the metal wall as these are strongly quenched in the experiment[25]. Note that the values for $F_p$ shown in Figure S8 are normalized by the Purcell enhancement due to vicinity of the SOI substrate which accounts for a factor of ≈ 1.8.

## 7. Polarization dependence of pump beam to waveguide coupling

The dependence of the gap in-coupling efficiency on the pump polarization is not only shown in simulations (shown in Figure S1) but can also be observed in the experiment. Figure S9 shows the dependence of the integrated emission signal on pump beam polarization at different positions on the sample and its comparison to the expected $\cos^2(\varphi)$ law, where $\varphi$ is the polarization angle. The pump wavelength is 980 nm, the same as in the measurements in the main text. A pump polarization of 0° corresponds to a polarization parallel to the gap (i.e. TM) and a pump polarization of 90° corresponds to a polarization perpendicular to the gap (i.e. TE).

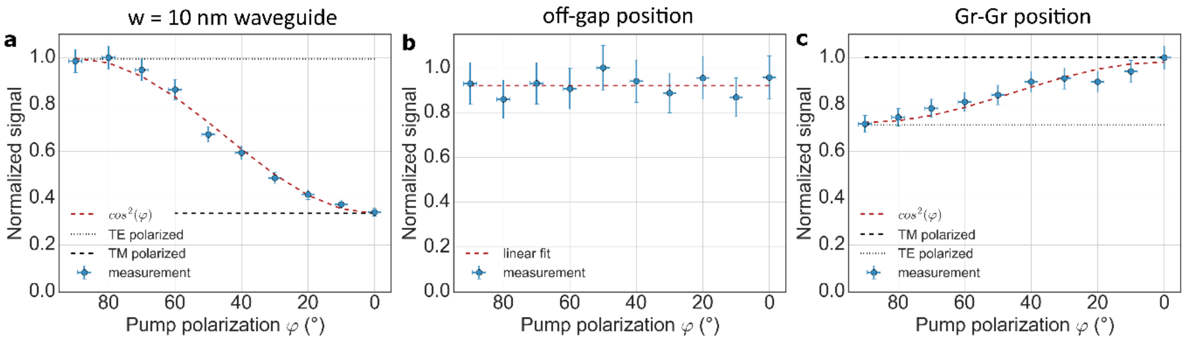


**Figure S9| Pump beam polarization (ϕ) measurements of integrated emission for different configurations at a wavelength of 980 nm. a** Central illumination of the Wg structure with a $w = 10$ nm gap waveguide. **b** off-gap position for reference (no gratings and no gap). **c** Central illumination of the grating-to-grating (Gr) configuration (no gap waveguide). The signal at a pump beam polarization perpendicular to the gap is labelled "TE polarized" and the signal at parallel polarization of the pump beam is labelled "TM polarized". Data points are the measurements while error bars were estimated to 5% for the waveguide and the Gr configuration and 10% of the off-gap position based on the standard deviation at the same power from the main manuscript.

Figure S9a shows the polarization dependence of the normalized integrated signal, and hence the in-coupled light into the gap, for a $w = 10$ nm hybrid gap waveguide. One observes that the measured signal drops by about 66% when a TM instead of a TE pump polarization is used. In accordance with the simulations shown above, we conclude that at least 66% of the collected signal originates from the ions illuminated in the gap waveguide alone. The rest of the collected signal stems from non-enhanced ions from outside of the waveguide gap. This is further underpinned by that fact that the same measurement at a position away from the plasmonic waveguide shows very little pump polarization dependence, as illustrated in Figure S9b. Moreover, the measurement in Figure S9c shows that 66% is a lower estimate on the proportion of gap luminescence, since in absence of the gap waveguide (i.e. the laser spot is centred between two focused gratings in "Gr-Gr" configuration) the TM polarized pump beam couples $\approx 30\%$ more efficiently to the gratings compared to the TE polarized pump. Thus, we estimate a signal contribution of erbium ions in the gap region to the entire detected signal in the Wg configuration to be larger than the fraction *f*=78% ($\approx 66\% + 30\% * 40\%$). The enhanced emission thus commands a larger proportion of the total emission, $f = 1 - S_{H,||}S_{Gr,\perp}/S_{Gr,||}S_{H,\perp}$, where $S_{H,j}$, $S_{Gr,j}$ are the signal values in parallel ($j = ||$) or perpendicular

($j$ =⊥) polarization to the gap of the gap waveguide and Gr-Gr device, respectively (Fig. S8). For the $w = 10\ nm$ gap waveguide, we find that a fraction $f_{pol} \geq 78\%$ of the collected signal from the Wg device originates from ions in the gap alone. Based on this information and the measurements of $I_{s,sat}$ for all other gap widths, listed in Table S1, we calculate the fractions ($f_{pol}$ values) for all other gap widths via $f_{pol,i} = \frac{I_{i-1}}{I_i} * (f_{i-1} - 1) + 1$. This expression results directly from the assumption that the signal contribution from ions in the non-gap region (i.e. the taper) stays constant for all measurements at different gap widths. Hence the difference between the saturation signals of two measurements determines all other $f_{pol}$ via $f_{pol,i} *\ I_i = I_i - I_{i-1} + I_{i-1} * f_{pol,i-1}$ with the start value $f_{pol,0} = 0.78$ and i = [10, 17, 30, 60]nm, i.e. the measured signal at the respective gap width $w = i$.

We underpin this estimate further by corroborating it with a second, independent measurement based on the closed gap reference sample described in the main manuscript (Fig. 3a,c). Here, $f_{closed}$ = 1 – ($I_{closed-gap}/I_i$) with i = [10, 17, 30, 60]nm and $I_i$ is the measured integrated signal at the respective gap width and $I_{closed-gap}$ the integrated signal for the same device with $w = 0$ (i.e. taper region only); both measured at 40 mW pump power . Both estimates for the respective fraction of the contributing signal from the gap are listed in Table 3. To provide a conservative estimate we use the values obtained from the polarization method ($f_{pol}$) to determine the gap signal $I_{gap}$ from the total measured signal $I_{Wg}$ used to determine EF as described in the main manuscript.

**Table 3| Signal contribution from the gap waveguide regions to the total collected signal in the Wg measurement.**

| Gap width $w$ | $f_{pol}$ (%) | $f_{closed}$ (%) |
|---|---|---|
| 10 nm | 78 | 88 |
| 17 nm | 64 | 79 |
| 30 nm | 50 | 71 |
| 60 nm | 33 | 64 |

## 8. Signal collection efficiency

This section explains how the collection efficiency ratio $\frac{\varepsilon_{c,Gr}}{\varepsilon_{c,Gap}}$ was estimated. Figure S10 shows a simplified illustration of the emission pathways of the ion luminescence in the Wg and the Gr-Gr configuration. The emitter is excited by linear polarized light (TE, perpendicular to the gap). The emission collected in the experiment can be estimated by considering two major pathways: i) fraction of light vertically radiated i.e. in normal direction off the sample ($X_{rad,a}$ in gap configuration

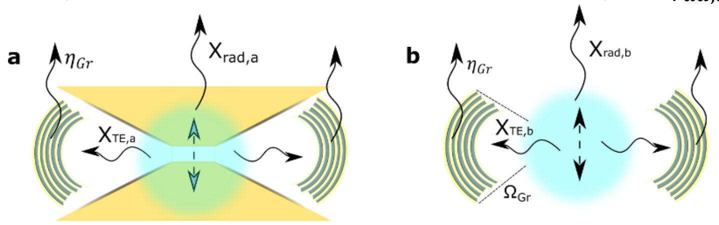


**Figure S10|** Sketch illustrating the emission pathways for in this case a polarized excited emitter (linear double arrow) in (a) the Wg configuration and (b) the Gr-Gr setting.

and $X_{rad,b}$ in Gr-Gr) where it is collected by an objective with a solid angle of ratio $\Omega_r$; ii) fraction of light coupled to the TE fundamental mode in both configurations ($X_{TE,a}$ for Wg and $X_{TE,b}$ for Gr-Gr) which propagates to gratings of collection efficiency $\eta_{Gr}$. The collection efficiency ratio of the two configurations can be written as:

$$\frac{\varepsilon_{c,Gr}}{\varepsilon_{c,Gap}} = \frac{X_{TE,b}\,\eta_{Gr}\,\Omega_{Gr}+X_{rad,b}\,\Omega_r}{X_{TE,a}\,\eta_{Gr}+\,\Omega_r\,X_{rad,a}} \tag{S8.1}$$

where the collection of light emitted by a horizontal dipole which is parallel to the pump polarization is estimated to $\Omega_{\mathrm{Gr}} = \frac{50°}{120°} \approx 42\%$, based on a 50° opening angle of each grating for the Gr-Gr configuration and the dipole emission angle in plane of around 120° as an upper estimate; a lower estimate for the coupling efficiency of ion emission from the gap to the TE slab mode (entirely collected by the gratings) is $X_{TE,a} \approx 80\%$ , extracted from experiments outlined in Figure S5b, as the focused grating couplers are designed to collect all the light exiting the gap, which however can be subject to slight reflections during the out-coupling from the gap; fraction of light coupled to the TE slab mode from the dipole $X_{TE,b} \approx 0.5 * 60\%$ = 30% where a TE/TM mode coupling ratio of 50:50 to the slab was conservatively estimated and 60% of dipole emission coupled to the slab, extracted from simulations; grating coupling efficiency $\eta_{Gr} \approx 30\%$ extracted from the experiment; the percentage of vertically emitted light from ions placed in a plain $SiO_2$ on Si system, 1 nm – 8 nm below the interface as described in the main text, is $X_{rad,b} \approx 20\%$ as extracted from simulations; the percentage of vertically emitted light out of the gap towards the objective for different gap width $\mathrm{w} = [10\text{ nm}, 17\text{ nm}, 30\text{ nm}, 60\text{ nm}]$ is $X_{rad,a}$= [0.03%, 0.04%, 0.24%, 0.24%]; The percentage of collected light by the objective (NA=0.4) used in the experiment is determined by the ratio of solid

angles while for $X_{rad,a}$ and $X_{rad,b}$ the simulation settings where such that approximately the entire emission in a half sphere was collected, resulting in $\Omega_r = A = sin^2\left(\frac{\theta_1}{2}\right) * (sin^2(\frac{\theta_0}{2}))^{-1}$ = 0.083, with $\theta_1 = 23.58°$ and $\theta_0 = 90°$; finally, we arrive at a lower bound estimate for the collection ratio of $\frac{\varepsilon_{C,Gr}}{\varepsilon_{C,Gap}} \geq 23\%$ based on Eq. (S8.1), which shows only a minor dependence on the gap width.

This lower bound estimate assumes a unity coupling efficiency from the dipole emission implanted below the plasmonic gap waveguide to the gap waveguide mode. This coupling efficiency can be quantified by the well-known β-factor, shown over the studied wavelength range in **Figure S11**.

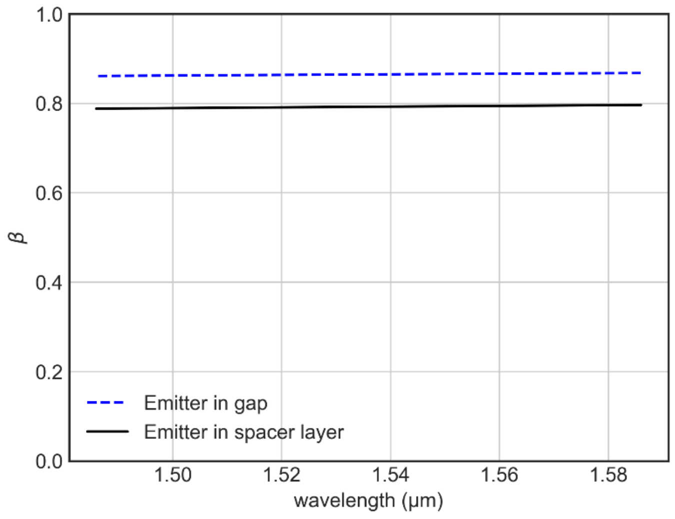


**Figure S11| Dipole to plasmonic waveguide mode coupling efficiency quantified by β.** Simulation results showing the broadband coupling of a dipole when positioned 1 nm below the gap (as studied in this work) and implanted into the gap waveguide.

Here we show dipole emission coupling to the gapmode of a 10 nm wide plasmonic waveguide. The emission coupling with emitters implanted in the $SiO_2$ spacer layer right below the gap (as in the experiment) shows a β-factor of ~80%. Here, emission channels other than the plasmonic waveguide are coupling to an in-plane mode at the oxide interlayer at the interface of the Au waveguide and the Si slab (~14% of loss), as well as quenching (i.e. excitation of higher order plasmonic modes). Light emitted into the substrate accounts for ~1% while upward radiation is minor (see above). For comparison we also show the case for an emitter implanted within the gap. The coupling efficiency can potentially be further increase by choosing a different refractive index within the gap (i.e. filling it with a higher index polymer). While the results are well in line with simulations results from the literature[26], experimental works show that even β-factors near unity are possible in plasmonic gap waveguides[27].

## 9. Purcell enhancement waveguide coupling of electric-dipole (ED) and magnetic-dipole (MD) emission

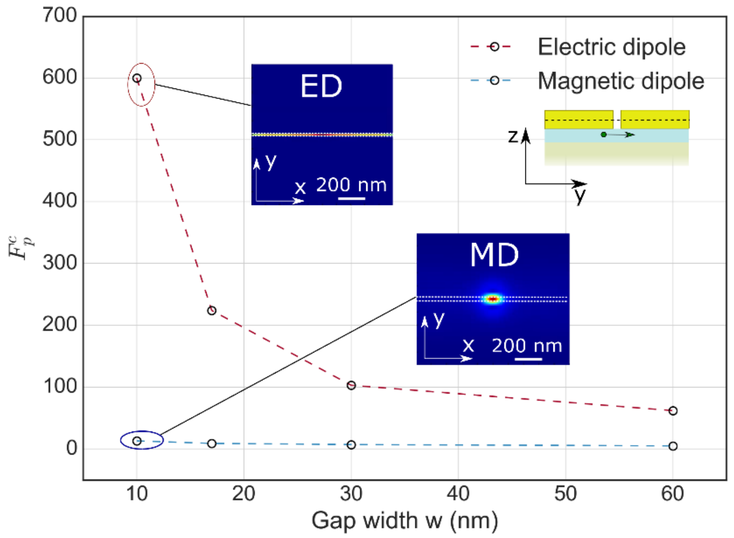


**Figure S12| Simulated average Purcell factor $F_p^c$ for an electric- and magnetic dipole emitter centrally positioned at $\Delta d =$ 1 nm below the plasmonic nanogap at λ = 1.536 µm**. The upper right inset shows the cross-section of the metal-insulator-metal (MIM) gap waveguide where the arrow indicates the dipole orientation. Here, the dashed black line indicates the position of the monitor which records the top view cross-sections of electric field $|E|$ due to the ED and MD coupling to the gap, shown on the left and on the bottom, respectively (both for $w = 10$ nm, white dotted lines mark the gap edges). The MD emission enhancement appears to be negligible in comparison to the ED emission enhancement and the MD emission does not couple efficiently to the predominantly transverse electric MIM gap mode.

## 10. Experimental setup

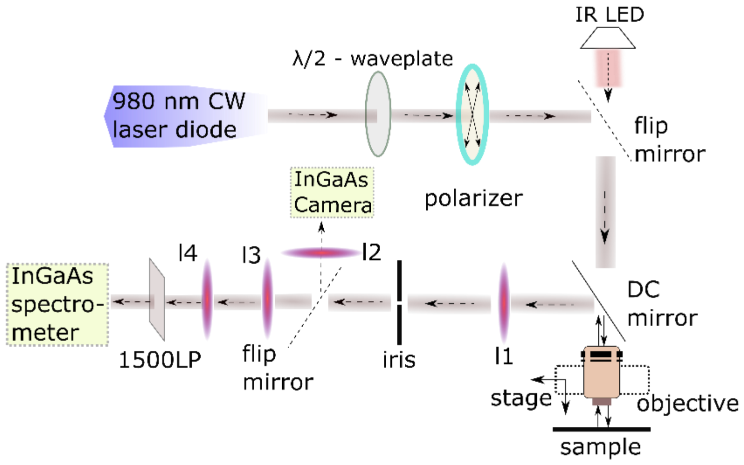


**Figure S13| Schematic of the experimental beamline** using an Agilent FPL4916 CW laser diode, a Mitutoyo infinity-corrected achromatic near-IR objective (NA=0.4, 20x, 2 cm WD) and a Princeton Instruments spectrometer (ActonSP2300) with a nitrogen cooled (−120°C) InGaAs 1D CCD detector array. For the spectrometer grating, a groove density of 600 gr/mm and a blaze wavelength of 1.6 µm with an efficiency of ≈85% (manufacturer information) at 1536 nm has been used. The lenses l1 - l4 collimate the beam onto the IR camera, the pinhole position, and the spectrometer. A dichroic short pass filter centred at 1180 nm (DCSP 1180) has been used, to allow the 980 nm CW pump to illuminate the sample while collecting the reflected signal from the sample at around 1536 nm. The pinhole/iris serves to isolate the signal of a single coupling grating from the rest of the sample.

## 11. Luminescence lifetime measurements

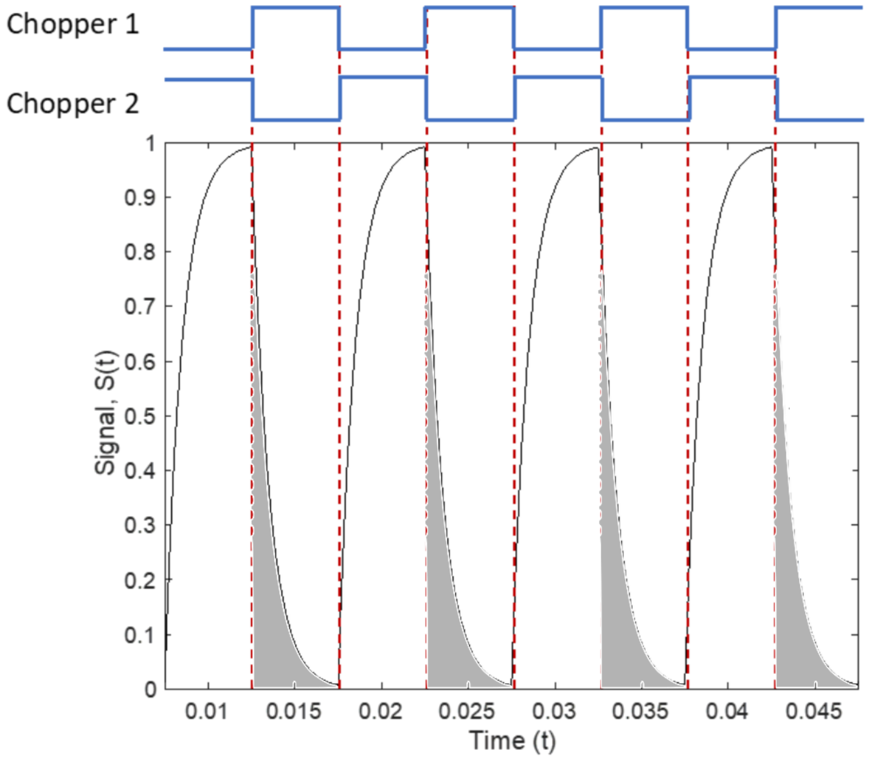


**Figure S14| Illustration of square wave modulated signal, $S(t)/S_0$, as a function of time for a modulation frequency of $\nu = 100$ Hz.** The modulation of the pump beam (chopper 1) and detected signal (chopper 2) are illustrated as $\pi$ out of phase. The shaded areas indicate where the detector integrates the signal leading to a value of $P_\pi/\nu t_{int}$ for each period.

The lifetimes of erbium ions implanted within the Gr-Gr control and 10 nm Wg devices were measured using a modulated pump and detection technique. Both the pump light and the luminescence were focused through mechanical choppers to produce near-square-wave responses, as shown in Figure S14. The excited state lifetime of erbium ions for square wave excitation at a frequency $\nu$ over a single period of modulation, $T = \nu^{-1}$, can be described by a three-level model. Assuming rapid transfer of electrons from the excited $^4\mathrm{I}_{11/2}$ state to the $^4\mathrm{I}_{13/2}$ state, and a low pump rate to avoid state saturation, a single differential equation may be used to describe the $^4\mathrm{I}_{13/2}$ population,

$$\frac{dN}{dt} = -\frac{N}{\tau} + R(t)N_T, \quad \text{(S11.1)}$$

where $R = r$ for $0 < t < T/2$, and $R = 0$ for $T/2 < t < T$. The emitted signal under continuous excitation is $S_0 = N/\tau = rN_T$. Under a modulated signal, it can be shown that, $S(t) = N(t)/\tau$,

$$S(t) = S_0\left(1 - \frac{e^{-t/\tau}}{(1+e^{-T/2\tau})}\right), \quad 0 < t < T/2 \quad \text{(S11.2)}$$

$$S(t) = S_0\frac{e^{-(t-T/2)/\tau}}{(1+e^{-T/2\tau})}, \quad T/2 < t < T, \quad \text{(S11.3)}$$

and this response is illustrated in Figure S14. A second chopper, phase-locked to the first, enables the signal to be measured over an integration time, $t_{int}$, with a phase shift, $\phi$, relative to the first modulation. This enables the lifetime to be spectrally resolved by using an imaging spectrometer. For a modulation frequency, $\nu$, the detector integrates over $\nu t_{int}$ periods. Assuming an experimental collection and detection signal of $\eta$, for out-of phase detection ($\phi = \pi$) we measure a signal,

$$P_\pi(\nu) = \eta \nu t_{int} S_0 \int_{T/2}^{T} \frac{e^{-(t-T/2)/\tau}}{(1+e^{-T/2\tau})} dt = \eta \nu \tau t_{int} S_0 \tanh((4\nu\tau)^{-1}). \qquad \text{(S11.4)}$$

To normalize this signal, we measure the sample luminescence without the two choppers over the same integration time, to find $P_* = \eta t_{int} S_0$. To determine the lifetime, we thus calculate

$$F(\nu) = \frac{P_\pi(\nu)}{P_*} = \nu\tau \tanh((4\nu\tau)^{-1}). \qquad \text{(S11.5)}$$

For a collection of emitters with a single exponential decay, the lifetime is returned for each measurement of $F(\nu)$. In the case of multi-exponential decay, the frequency resolved response is necessary to correctly distinguish the various decay components. Note that for $\nu\tau \ll 1$, $\tanh((4\nu\tau)^{-1}) \approx 1$ , a linear variation is found, $\nu\tau \approx F(\nu)$, which provides a fixed frequency link to the lifetime.

**Multi-exponential decay**

For mixtures of emitters with differing lifetimes, we will find an average signal,

$$P_\pi(\nu) = P_* \sum_i^N a_i \, \nu\tau_i \tanh((4\nu\tau_i)^{-1}) \qquad \text{(S11.6)}$$

where $a_i$ are the proportions of emitters with lifetimes, $\tau_i$, and $\sum_i a_i = 1$. In the bi-exponential case with $a_2 = 1 - a_1$

$$P_\pi(\nu) = P_* \nu [a_1 \tau_1 \tanh((4\nu\tau_1)^{-1}) + (1 - a_1)\tau_2 \tanh((4\nu\tau_2)^{-1})]. \qquad \text{(S11.7)}$$

This is the situation observed in our experiments. There are thus three parameters to find to determine the variation of $P_\pi(\nu)/P_*$. Note also that there are three regimes, which provide information about the mixture of emitters and their lifetimes:

| $\nu\tau_1, \nu\tau_2 \ll 1$ | $\nu\tau_1 > 1, \nu\tau_2 \ll 1$ | $\nu\tau_1, \nu\tau_2 > 1$ |
|---|---|---|
| $\frac{P_\pi(\nu)}{P_*} = \nu(a_1\tau_1 + a_2\tau_2)$ | $\frac{P_\pi(\nu)}{P_*} = \frac{a_1}{4} + (1 - a_1)\nu\tau_2$ | $\frac{P_\pi(\nu)}{P_*} = \frac{1}{4}$ |

These formulae explain the data observed in experiments on the Gr-Gr control and 10 nm gap Wg samples described in the main text. Figure S15 shows the experimental data with the theoretical fits to these data.

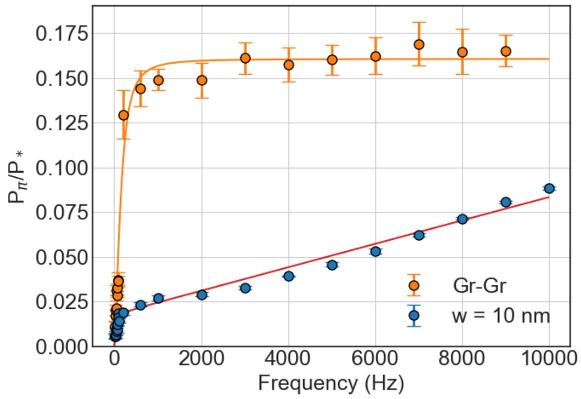


**Figure S15| Frequency dependency of the ratio of modulated to unmodulated power detected for the Gr-Gr and 10 nm gap devices.** Both devices were excited with a 980 nm CW laser. Solid lines are the fitted bi-exponential decay functions Eq. (S8.7). Data points are the mean and error bars the standard deviation.

To cover the span of the modulation frequency range, a dual frequency blade and a chopper with 100 slots were used. The dual frequency blade was used to generate the low modulation frequency from 2 Hz to 100 Hz, while the second chopper was used to generate high modulation frequency from 200 Hz to 10 kHz.

**Experimental parameters:**

The luminescence of the Gr-Gr control device without modulation $P_*$ was measured, by exciting the sample with a 980 nm CW laser at the power of 40 mW over the integration time of 30 seconds. The out of phase luminescence signal $P_\pi$ was measured by using the same pump power and integration with both choppers on. The Wg sample was measured by using a lower pump power of 20 mW. The other conditions were identical to the Gr-Gr control device measurement.

**Fitting parameters:**

The signal $P_\pi\ (\nu)\ /P_*$ as a function of the modulation frequency $\nu$ was fitted by using the bi-exponential decay function

$$P_\pi(\nu) = P_*\nu[a_1\tau_1\tanh((4\nu\tau_1)^{-1}) + (1-a_1)\tau_2\tanh((4\nu\tau_2)^{-1})]. \qquad \text{(S11.8)}$$

The fitting parameters of the Gr-Gr sample are $a_1 = 0.643$, $\tau_1 = 1$ ms, $\tau_2 = 5$ ns.

The parameters of the 10 nm gap sample are $a_1 = 0.071$, $\tau_1 = 2.5$ ms, $\tau_2 = 7.1$ µs.

**Measuring the power dependence of lifetime.**

Generally, the lifetime will depend on the pump power. Hence, the natural lifetime results from extrapolation to the value at zero-pump power (see Figure 3f). Once the modulation frequency dependence of the emitters was determined, the lifetime could be extracted at a fixed modulation frequency as a function of the pump power, $r$. For a single exponential decay, we can use the following relationship:

$$\nu \frac{P_*(r)}{P_\pi(r)} \approx \tau(r)^{-1}. \quad \text{(S11.9)}$$

For a bi-exponential decay, the same approach can be used. For the slower emitters, $\nu\tau_1 \ll 1, \tau_1 \gg \tau_2$,

$$\nu a_1 \frac{P_*(r)}{P_\pi(r)} \approx \tau_1(r)^{-1}. \quad \text{(S11.10)}$$

The power dependence of the faster emitters in the regime $\nu\tau_1 > 1, \nu\tau_2 \ll 1$, can also be determined using the expression:

$$\frac{(1-a_1)\nu}{\frac{P_\pi(r)}{P_*(r)} - \frac{a_1}{4}} = \tau_2(r)^{-1}. \quad \text{(S11.11)}$$

Such measurements were conducted on the Gr-Gr and 10 nm gap Wg devices at fixed frequency as a function of pump power, as shown in Figure 3f of the main text. This data shows a linear dependency of the emission rates on pump power.

**Spectrally resolved lifetime**

This technique allows rapid collection of spectrally resolved lifetime data by using an imaging spectrometer. Figure S16 shows the spectrally resolved lifetime of erbium ions from the Gr-Gr sample (Δλ =20 nm binning). Due to the weak signal from this sample, the spectral resolution is

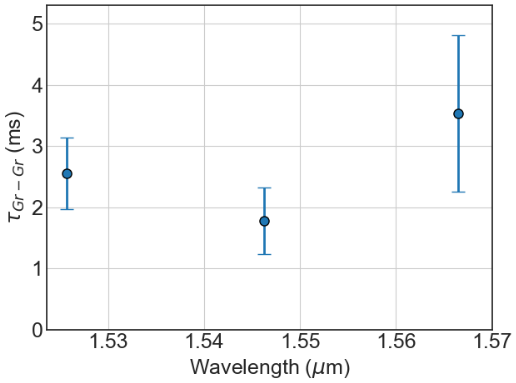


**Figure S16| Spectrally resolved lifetime of erbium ions from the Gr-Gr sample.** The lifetime is extracted from the power dependent emission rate measurements, with excitation power from 5 mW to 40 mW. To ensure good signal to noise ratio, spectral resolution of the lifetime is set to be around 20 nm. Data points are the mean and error bars the standard deviation.

limited. Nonetheless, the erbium lifetime is uniform across the erbium emission band with an average lifetime of $2.62\ \mathrm{ms} \pm 0.72\ \mathrm{ms}$. The spectrally resolved enhancement factor (lifetime ratio) for the 10 nm gap Wg sample is shown in Figure 3e of the main text.

## 12. Power dependence of the signal spectrum

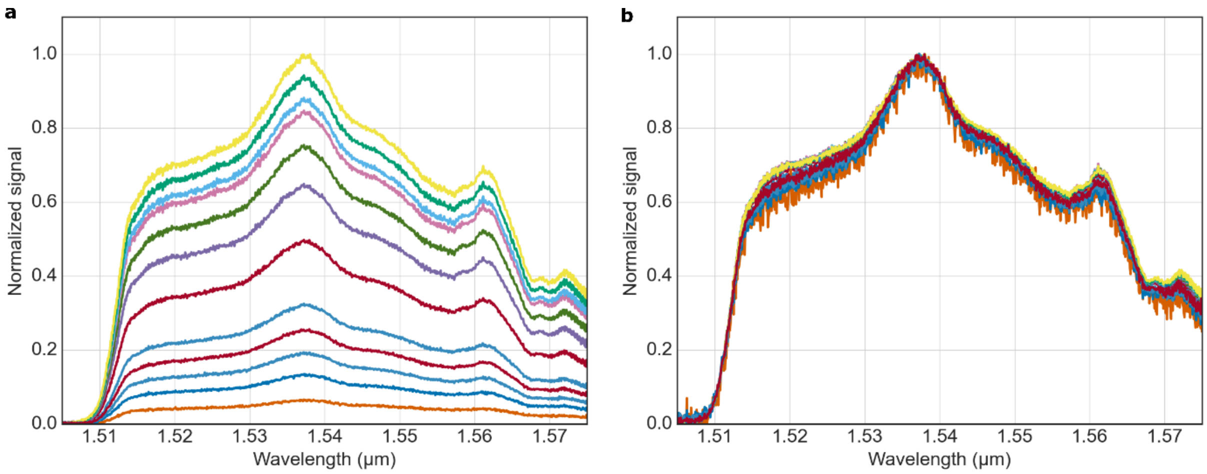


**Figure S17| Normalized luminescence spectra** measured at the pump power $[2, 4, 6, 8, 10, 15, 20, 25, 30, 35, 40]$ mW (from orange to yellow color-coding) for a waveguide structure with a $w = 10$ nm gap. **a**, Normalized to the overall maximum signal and **b**, normalized to the maximum signal of each spectrum. No pump power-dependent red-shift or change of the spectral shape is observed, which excludes any form of self-stimulated emission.

### Supplementary References

1. Jun, Y. C., Pala, R. & Brongersma, M. L. Strong modification of quantum dot spontaneous emission via gap plasmon coupling in metal nanoslits. *Journal of Physical Chemistry C* **114**, 7269–7273 (2010).

2. Fan, P., Huang, K. C. Y., Cao, L. & Brongersma, M. L. Redesigning photodetector electrodes as an optical antenna. *Nano Lett* **13**, 392–396 (2013).

3. Ziegler, J. F. & Biersack, J. P. The Stopping and Range of Ions in Matter. in *Treatise on Heavy-Ion Science* 93–129 (Pergamon Press, New York, 1985).

4. Ziegler, J. F. Interactions of ions with matter. www.srim.org (2021).

5. Lu, Y.-W. *et al.* Erbium diffusion in silicon dioxide. *Appl Phys Lett* **97**, 141903 (2010).

6. Szeles, C., Nielsen, B., Asoka-Kumar, P. & Lynn, K. G. Role of implantation-induced defects in surface-oriented diffusion of fluorine in silicon. **76**, 3403 (1994).

7. Nielsen, M. P., Shi, X., Dichtl, P., Maier, S. A. & Oulton, R. F. Giant nonlinear response at a plasmonic nanofocus drives efficient four-wave mixing. *Science (1979)* **358**, 1179–1181 (2017).

8. Keck, D. B. & Tynes, A. R. Spectral Response of Low-Loss Optical Waveguides. *Appl Opt* **11**, 1502 (1972).

9. Güsken, N. A., Nielsen, M. P., Nguyen, N. B., Maier, S. A. & Oulton, Rupert. F. Nanofocusing in SOI-based hybrid plasmonic metal slot waveguides. *Opt Express* **26**, 30634 (2018).

10. Mertens, H. & Polman, A. Plasmon-enhanced erbium luminescence. *Appl Phys Lett* **89**, (2006).

11. Polman, A. Erbium implanted thin film photonic materials. *J Appl Phys* **82**, 1–39 (1997).

12. Laming, R. I. *et al.* Optimal pumping of erbium-doped-fibre optical amplifiers. *IEE Conference Publication* 25–28 (1988).

13. Barnes, W. L., Morkel, P. R., Reekie, L. & Payne, D. N. High-quantum-efficiency Er^3+ fiber lasers pumped at 980 nm. *Opt Lett* **14**, 1002 (1989).

14. Hu, Y. *et al.* Numerical analyses of the population dynamics and determination of the upconversion coefficients in a new high erbium-doped tellurite glass. *Journal of the Optical Society of America B* **18**, 1928 (2001).

15. Barmenkov, Y. O., Kir'yanov, A. V., Guzmán-Chávez, A. D., Cruz, J.-L. & Andrés, M. V. Excited-state absorption in erbium-doped silica fiber with simultaneous excitation at 977 and 1531 nm. *J Appl Phys* **106**, 083108 (2009).

16. Blixt, P., Nilsson, J., Carlnas, T. & Jaskorzynska, B. Concentration-Dependent Upconversion in Er 3+-Doped Fiber Amplifiers: Experiments and Modeling. *IEEE Transactions Photonics Technology Letters* **3**, 996–998 (1991).

17. Garrido, B. *et al.* Excitable Er fraction and quenching phenomena in Er-doped SiO2 layers containing Si nanoclusters. *Phys Rev B* **76**, 245308 (2007).

18. Van Den Hoven, G. N. *et al.* Upconversion in Er-implanted Al2O3 waveguides. *J Appl Phys* **79**, 1258–1266 (1996).

19. Snoeks, E., Lagendijk, A. & Polman, A. Measuring and Modifying the Spontaneous Emission Rate of Erbium near an Interface. *Phys Rev Lett* **74**, 2459–2462 (1995).

20. Quimby, R. S. Output saturation in a 980-nm pumped erbium-doped fiber amplifier. *Appl Opt* **30**, (1991).

21. Quimby, R. S., Miniscalco, W. J. & Thompson, B. Clustering in erbium-doped silica glass fibers analyzed using 980 nm excited-state absorption. *J Appl Phys* **76**, 4472–4478 (1994).

22. Snoeks, E. *et al.* Cooperative upconversion in erbium-implanted soda-lime silicate glass optical waveguides. *Journal of the Optical Society of America B* **12**, 1468 (1995).

23. Philippe M. Becker, Anders A. Olsson, Jay R. Simpson, and J. R. S. *Erbium-Doped Fiber Amplifiers : Fundamentals and Technology*. (Elsevier Science & Technology, San Diego, 1999).

24. Novotny, L. & Hecht, B. *Principle of Nano-optics*. (Cambridge University Press, 2006).

25. Anger, P., Bharadwaj, P. & Novotny, L. Enhancement and Quenching of Single-Molecule Fluorescence. *Phys Rev Lett* **96**, 113002 (2006).

26. Jun, Y. C., Kekatpure, R. D., White, J. S. & Brongersma, M. L. Nonresonant enhancement of spontaneous emission in metal-dielectric-metal plasmon waveguide structures. *Phys Rev B Condens Matter Mater Phys* **78**, 1–4 (2008).

27. Fu, M. *et al.* Near-unity Raman β-factor of surface-enhanced Raman scattering in a waveguide. *Nat Nanotechnol* **17**, 1251–1257 (2022).
